# Pediatric anxiety and daily fine particulate matter: A longitudinal study

**DOI:** 10.1016/j.psycom.2022.100077

**Published:** 2022-10-09

**Authors:** Andrew Vancil, Jeffrey R. Strawn, Erika Rasnick, Amir Levine, Heidi K. Schroeder, Ashley M. Specht, Ashley L. Turner, Patrick H. Ryan, Cole Brokamp

**Affiliations:** a Cincinnati Children’s Hospital Medical Center, Division of Biostatistics and Epidemiology, USA; b University of Cincinnati, Anxiety Disorders Research Program, Department of Psychiatry & Behavioral Neuroscience, USA; c Department of Psychiatry, College of Physicians and Surgeons, Columbia University, USA; d Division of Child and Adolescent Psychiatry, New York State Psychiatric Institute, USA; e University of Cincinnati, College of Medicine, Department of Pediatrics, USA

## Abstract

Daily variations in ambient fine particulate matter (PM_2.5_) could contribute to the morbidity of anxiety disorders in children and adolescents, but has not yet been studied longitudinally at a daily level. We tested this association using repeated weekly measures of anxiety symptom severity in a group of 23 adolescents with generalized anxiety disorder. After estimating ambient PM_2.5_ concentrations using a validated model, we found that increased concentrations were significantly associated with increased anxiety symptom severity and frequency two, three, and four days later. PM_2.5_ may be a novel, modifiable exposure that could inform population level interventions to decrease psychiatric morbidity.

## 1. Background

Anxiety disorders are a cause of major morbidity in children and adolescents, often interfering with development and education (Institute of Medicine (U.S, 2009; Erskine et al., 2015), but also carry an increased long-term risk of mood disorders, substance use disorders, and suicide well into adulthood (Weissman et al., 1999; Pine et al., 1998; Fergusson and Woodward, 2002; Kim-Cohen et al., 2003; Caspi et al., 1996; Bittner et al., 2007; Woodward and Fergusson, 2001; Beesdo et al., 2007; Asselmann et al., 2014). Although our understanding of the role of genetic, family environment, temperament, cognition and psychosocial milieu in the development of anxiety disorders has increased (Kieling et al., 2011; Reiss, 2013), few modifiable risk factors have been identified at a population level (Guloksuz et al., 2018). One potential such contributor to psychiatric morbidity of anxiety disorders is outdoor air pollution, specifically ambient particulate matter with an aerodynamic diameter smaller than 2.5 μm (PM_2.5_). Importantly, PM_2.5_ may lead to anxiety disorders by inducing oxidative stress and inflammation in the central nervous system, including microglial activation and toxicity in the brain (Block and Calderon-Garciduenas, 2009; Block et al., 2012; Calderon-Garciduenas et al., 2002; Costa et al., 2014; Allen et al., 2017).

Epidemiologic studies have used emergency department (ED) utilization data within daily time series and case-crossover studies to show that short term increases in ambient air pollution are associated with the exacerbation of psychiatric disorders (Kim et al., 2019; Oudin et al., 2018; Qiu et al., 2019), stress-related disorders (Kim et al., 2019), depressive disorders (Szyszkowicz et al., 2009, 2016; Wang et al., 2018; Cho et al., 2014), suicide attempts (Szyszkowicz et al., 2010), and completed suicide (Bakian et al., 2015). Though several studies have linked recent (30–180 days) PM_2.5_ with increased anxiety and depression symptom severity and frequency in adults (Power et al., 2015; Pun et al., 2017), acute (1–3 days) exposures have not yet been studied outside of ED utilization data. PM_2.5_ has also been associated with anxiety and related symptoms in pediatric populations, including psychiatric ED visits (Leeb et al., 2020). In a sample of children and adolescents presenting to the emergency department for psychiatric complaints, increased daily PM_2.5_ was related to a higher likelihood of visits related to anxiety and suicidal ideation. Moreover, this was especially true for children living in areas of high community material deprivation who are more susceptible to the effects of PM_2.5_ (Brokamp et al., 2019).

Here, we sought to determine the relationship between acute ambient PM_2.5_ exposures and repeated measures of anxiety symptom severity.

## 2. Material and methods

### 2.1. Study population

The study population was composed of 23 adolescents, aged 12–17 years with a primary diagnosis of generalized anxiety disorder (GAD), from the Cincinnati, OH, USA area enrolled in the placebo arm of a randomized controlled trial performed between 2015 and 2018 (Strawn et al., 2020). Adolescents included in the trial met *DSM-IV-TR* criteria for GAD, had a Pediatric Anxiety Rating Scale (PARS) score ≥15, and a Clinical Global Impression—Severity of Illness (CGI-S) score ≥4, as previously described, did not have any significant co-morbidities, and were not taking any psychotropic medications (Strawn et al., 2020).

### 2.2. Assessment of anxiety severity

The Pediatric Anxiety Rating Scale (PARS), is a clinician-administered, validated instrument used to assess pediatric anxiety severity in clinical trials of anxious youth (The Pediatric Anxiety Rating Scale, 2002). A 50-item checklist was rated by a board-certified child and adolescent psychiatrist with established reliability on this measure followed by a global assessment of seven dimensions of severity using a 0 to 5 scale. Of the seven dimensions of PARS assessed, we used the clinical trials scoring which sums five (symptom frequency, severity of distress, avoidance, interference at home, interference out of home), for a possible score range of 0–25 (Walkup et al., 2001). Anxiety symptom severity was assessed serially (about once a week) for each patient over the course of eight weeks.

### 2.3. Exposure and confounder assessment

Participant geocoded residential addresses were used to estimate daily ambient concentrations of average PM_2.5_ via a previously validated spatiotemporal exposure assessment model (Brokamp, 2022). The model was derived using meteorologic data, industrial PM_2.5_ emissions data, and spatiotemporal PM_2.5_ interpolation measures all calibrated with ground based PM_2.5_ monitoring data. Within the study region the prediction model was highly accurate, with a cross validated R^2^ of 0.92 and a median absolute error of 1.00 μg/m^3^. The geocoded addresses were also used to derive daily average air temperature and relative humidity from the North American Regional Reanalysis (NARR) database (Mesinger et al., 2006).

### 2.4. Statistical analysis

To identify the relationship between daily PARS and exposures to PM_2.5_ over time, we utilized a distributed lag nonlinear model (DLNM) framework. DLNMs, generally, use past and current values as predictors, referred to as “lagged” values to help identify temporal windows when the relationship between an exposure and outcome occurs (Gasparrini et al., 2010). We considered the binary logarithm of daily PM_2.5_ estimated exposures from the day of each PARS assessment and the previous six days. The relationship was modeled using a natural cubic spline, with 3 degrees of freedom, for both the dose-response and lag-response relationship. We considered a non-linear dose-response relationship because supralinear relationships, in which the risk of adverse health outcomes increases at a greater rate at lower exposure concentrations than when compared to higher concentrations, has been previously observed for PM_2.5_ and other health outcomes(Xie et al., 2015). To adjust for confounding by other temporal factors related to both PARS and PM_2.5_, we included natural cubic splines, with 3 degrees of freedom, of air temperature, relative humidity, and day of the year. We excluded day of the week as a temporal confounder because we did not observe any significant differences of PM_2.5_ concentrations or PARS scores across different days of the week. We used a fixed effects regression model by including subject-specific intercepts, as in the case time series design (Gasparrini, 2021), to avoid confounding by characteristics that do not vary throughout the study period (e.g., socioeconomic status). Sensitive windows were identified by plotting predicted changes in PARS at each lag day for a hypothetical change from the 25th percentile to the 50th and 75th percentiles of PM_2.5_ concentrations. All statistical computing and analyses were done using R (Team, 2020), specifically the dlnm (Gasparrini et al., 2017) package.

## 3. Results

Study participants (n = 23) were 74% female with a mean age of 15.0 (range (Kieling et al., 2011; Calderon-Garciduenas et al., 2002)). Each participant contributed a median of 6 PARS scores, for a total of 123 within the eight weeks (range (Erskine et al., 2015; Caspi et al., 1996)), mostly occurring one week apart. The average PARS score for all participants was 16 (standard deviation: 3.89). We estimated average PM_2.5_ for each PARS score assessment on each of the seven days leading up to (and including) the day the score was ascertained, for a total follow up of 861 person-days. We calculated a mean 24-h average PM_2.5_ of 8.35 μg/m^3^ (minimum: 2.22, 25th percentile: 5.93, median: 7.96, 75th percentile: 10.46, maximum: 26.38 μg/m^3^).

The estimated non-linear associations between PM_2.5_ and PARS scores from our adjusted regression model are shown in the figure. Fig. 1A shows that a change in PM_2.5_ concentration from the 25th percentile to the median (5.93 μg/m^3^ versus 7.96 μg/m^3^) was significantly associated with increased PARS scores two (0.57, 95% CI: 0.06, 1.07), three (0.82, 95% CI: 0.18, 1.45), and four (0.62, 95% CI: 0.11, 1.14) days later. Fig. 1B shows the estimated change in PARS score according to a range of PM_2.5_ concentrations (as compared to the 25th percentile, 5.93 μg/m^3^) for a three-day lag period. Here, there is visual evidence of a supralinear dose-response curve, with increases at lower concentrations being associated with larger changes in PARS scores compared to similar increases at higher concentrations. When considering a greater change in exposure from the 25th percentile to the 75th percentile (5.93 μg/m^3^ versus 10.46 μg/m^3^), the average PARS scored significantly increased three (1.24, 95% CI: 0.17, 2.31) and four (0.97, 95% CI: 0.10, 1.83) days later.

## 4. Discussion

The association between PM_2.5_ and PARS occurred independently of both (1) characteristics that do not vary for an individual within the follow-up period, such as socioeconomic status, race, or sex and (2) measured exposures that do vary for an individual within the follow-up period and could confound the association, such as temperature and humidity. While a 1.24-point increase in PARS score may seem like a relatively small individual-level change, because nearly everyone is exposed to ambient PM_2.5_, it can have a large impact at a population-level.

One advantage of our study was the use of the Pediatric Anxiety Risk Score (PARS) because it is a clinician-administered, structured instrument that is often considered the gold standard for assessing anxiety severity in children and adolescents. Unlike other instruments, such as the Generalized Anxiety Disorder 7-item (GAD-7) (Mossman et al., 2017), PARS has more dimensionality and captures impairment. However, the PARS captures symptom severity during the previous seven days and may not be precise enough to capture daily temporal relationships. Future studies could work to develop an instrument for assessing anxiety symptom severity that may better capture daily fluctuations in anxiety symptoms useful for studying associations with exposures that have high temporal variation. It is important to consider that this study was conducted in adolescents with severe anxiety and may not be generalizable to populations in which severe anxiety symptoms are less common or non-existent.

The finding that the CNS may be particularly sensitive to daily fluctuating levels of PM_2.5_ also avails the possibility of tailoring interventions for the treatment and prevention of anxiety beyond standard treatments (e.g., cognitive behavioral therapy and selective serotonin reuptake inhibitors [SSRIs]). Reducing air pollution exposure can be achieved through primary interventions and policy changes. Notably, the associations we found here occurred when studying PM_2.5_ exposures that did not exceed the Environmental Protection Agency’s National Ambient Air Quality Standard for daily PM_2.5_ of 35 μg/m^3^. As the climate changes and wildfires increase in frequency and severity, short term periods of very high PM_2.5_ exposure exceeding these standards are expected to also increase in frequency and severity(Liu et al., 2016; Di Virgilio et al., 2019; Neumann et al., 2021). In conclusion, fine particulate matter may be a novel, modifiable influence that could be used in the future at a population level to intervene and protect adolescents from the effects of psychiatric disease morbidity.

## Figures and Tables

**Fig. 1A.**
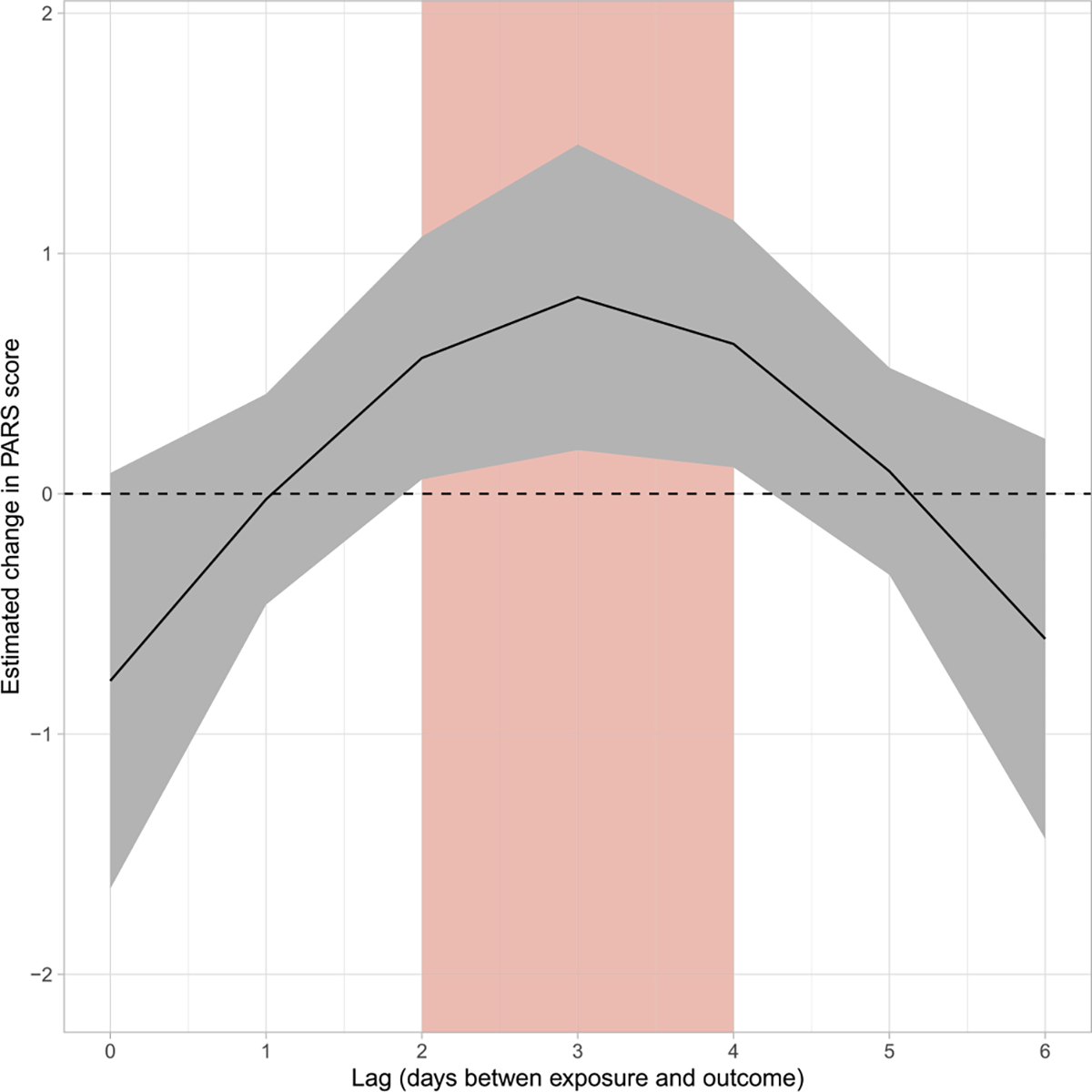
Estimated change in PARS score by lag associated with an increase in PM_2.5_ from the 25th percentile to the median (5.93 versus 7.96 μg/m^3^). The red, shaded rectangle denotes lag periods where the estimated change in PARS score was significantly different from zero.

**Fig. 1B.**
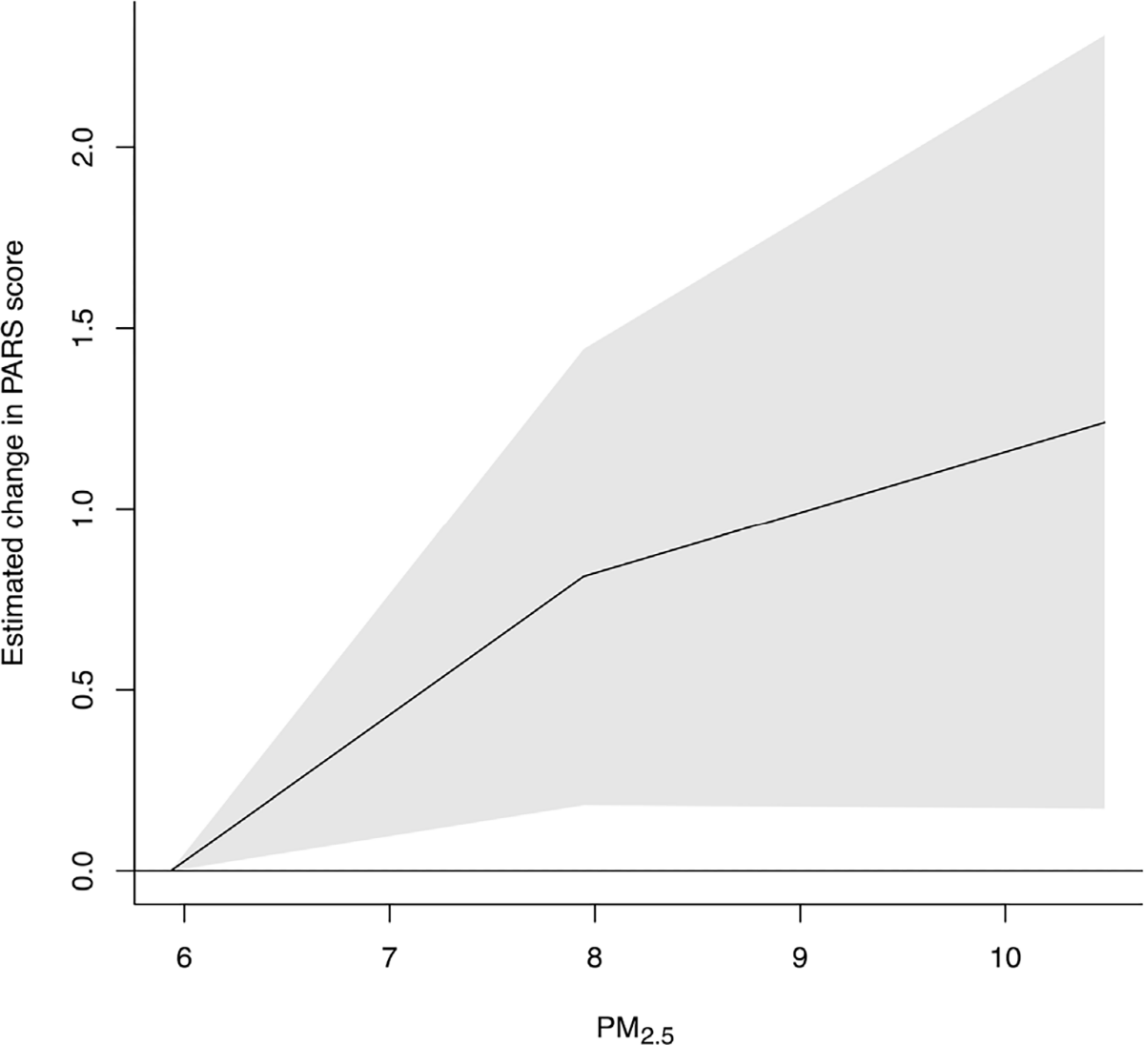
Dose-response curve for the estimated change in PARS score three days after an increase in PM_2.5_ concentrations relative to the 25th percentile (5.93 μg/m^3^). The range of the x-axis is from the 25th to the 75th percentile of PM_2.5_ concentrations.
